# Life history and habitat do not mediate temporal changes in body size due to climate warming in rodents

**DOI:** 10.7717/peerj.9792

**Published:** 2020-09-24

**Authors:** Aluwani Nengovhela, Christiane Denys, Peter J. Taylor

**Affiliations:** 1 South African Research Chair in Biodiversity Value and Change and Centre for Invasion Biology, School of Mathematical and Natural Sciences, University of Venda, Thohoyandou, Limpopo, South Africa; 2 Institut de Systématique, Evolution, Biodiversité (ISYEB), UMR 7205, CNRS, MNHN, UPMC, EPHE, Sorbonne Universités, Paris, France; 3 Zoology and Entomology Department and Afromontane Research Unit, University of the Free State, QwaQwa Campus, Phuthaditjhaba, South Africa

**Keywords:** Morphology, Body size, Rodentia, Africa, Climate change, Museum specimen, Temporal changes, Bergmann’s rule, Skull morphometrics

## Abstract

Temporal changes in body size have been documented in a number of vertebrate species, with different contested drivers being suggested to explain these changes. Among these are climate warming, resource availability, competition, predation risk, human population density, island effects and others. Both life history traits (intrinsic factors such as lifespan and reproductive rate) and habitat (extrinsic factors such as vegetation type, latitude and elevation) are expected to mediate the existence of a significant temporal response of body size to climate warming but neither have been widely investigated. Using examples of rodents, we predicted that both life history traits and habitat might explain the probability of temporal response using two tests of this hypothesis. Firstly, taking advantage of new data from museum collections spanning the last 106 years, we investigated geographical and temporal variation in cranial size (a proxy for body size) in six African rodent species of two murid subfamilies (Murinae and Gerbillinae) of varying life history, degree of commensality, range size, and habitat. Two species, the commensal *Mastomys natalensis,* and the non-commensal *Otomys unisulcatus* showed significant temporal changes in body size, with the former increasing and the latter decreasing, in relation with climate warming. Commensalism could explain the increase in size with time due to steadily increasing food availability through increased agricultural production. Apart from this, we found no general life history or habitat predictors of a temporal response in African rodents. Secondly, in order to further test this hypothesis, we incorporated our data into a meta-analysis based on published literature on temporal responses in rodents, resulting in a combined dataset for 50 species from seven families worldwide; among these, 29 species showed no significant change, eight showed a significant increase in size, and 13 showed a decline in size. Using a binomial logistic regression model for these metadata, we found that none of our chosen life history or habitat predictors could significantly explain the probability of a temporal response to climate warming, reinforcing our conclusion based on the more detailed data from the six African species.

## Introduction

The widespread effects of anthropogenic climate warming on biodiversity and ecosystems are undeniable, with different responses reported. These include phenological changes (e.g., Diamond et al., 2011; Miller-Rushing & Primack, 2008), species distributional range shifts (e.g., Hickling et al., 2006; Monadjem et al., 2013; Parmesan & Yohe, 2003; Taylor et al., 2015; Taylor et al., 2016; Walther et al., 2002) and decreasing body size (e.g., Daufresne, Lengfellner & Sommer, 2009; Gardner et al., 2011; Sheridan & Bickford, 2011). The above-mentioned responses are regarded collectively as the universal ecological responses to climate warming (Daufresne, Lengfellner & Sommer, 2009; Ohlberger, 2013).

Recently, morphological responses to climate change (usually entailing body size) have received growing interest, with changes reported in both aquatic and terrestrial environments (Gardner et al., 2011; Sheridan & Bickford, 2011). Body size varies geographically and temporally within species (Alhajeri & Steppan, 2016; McNab, 2010; Nengovhela, Baxter & Taylor, 2015; Stumpp, Fuzessy & Paglia, 2016; Yom-Tov & Geffen, 2011; Yom-Tov & Yom-Tov, 2004; Yom-Tov & Yom-Tov, 2005; Yom-Tov & Yom-Tov, 2012; Yom-Tov et al., 2006) in response to environmental variables including ambient temperature and precipitation (Bergmann, 1847; Blackburn, Gaston & Loder, 1999; Blois, Feranec & Hadly, 2008; James, 1970), food availability (McNab, 2010; Yom-Tov & Yom-Tov, 2004; Yom-Tov & Yom-Tov, 2005; Yom-Tov, Yom-Tov & Baagoe, 2003; Yom-Tov et al., 2010a), predation regimes (Gosler, Greenwood & Perrins, 1995), habitat fragmentation (Schmidt & Jensen, 2003) and competition (Meiri, Yom-Tov & Geffen, 2007; Raia & Meiri, 2006). Different mechanisms have been advanced to explain these effects, most commonly, in a geographic context (due to altitude and latitude), Bergmann’s Rule, which states that individuals under warmer climates should be smaller in body size as compared to individuals in colder climates (Bergmann, 1847; Mayr, 1956). While this rule was originally formulated at an interspecific pattern within genera, by far, most studies, including metanalyses, test this rule at the intraspecific level (Alhajeri & Steppan, 2016). Bergmann’s Rule or related hypotheses that explain inverse temperature-body size clines in endotherms and ectotherms (termed “Bergmann’s clines” hereafter) have been invoked to explain body size changes due to global climate warming in a range of animals including insects, birds, rodents and salamanders (Babin-Fenske, Anand & Alarie, 2008; Blanckenhorn, 2015; Caruso et al., 2014; McCoy, 2012; Nengovhela, Baxter & Taylor, 2015). However, the authenticity, general applicability and ultimate causation of Bergmann’s clines have been questioned (Calder, 1984; Gardner et al., 2011; McNab, 1971; McNab, 2010; Millien et al., 2006; Scholander, 1955; Teplitsky & Millien, 2014; Yom-Tov & Geffen, 2006). Studies contradicting Bergmann’s clines have been reported, showing an increase in size with increasing temperature, either spatially or temporally, e.g., Norwegian and Swedish otters (*Lutra lutra*), Alaskan masked shrews (*Sorex cinereus*), Japanese mice (*Apodemus speciosus*) and red foxes (*Vulpes vulpes*) (Yom-Tov & Yom-Tov, 2004; Yom-Tov & Yom-Tov, 2005; Yom-Tov & Yom-Tov, 2012; Yom-Tov et al., 2006; Yom-Tov et al., 2010a). Reasons given for such increases in size with time included habitat factors such as improved food availability and urbanization and physiological factors such as reduced energy expenditure (Yom-Tov & Yom-Tov, 2005; Yom-Tov et al., 2006; Yom-Tov et al., 2010a).

Life history traits (those relating to survival and reproduction of an organism) have been shown to have a direct link to fitness (Lloyd, 1987), as an organism’s success depends on the ability to grow to a reproductive age, reproductive output and timing of reproduction (Moravcová et al., 2015). Body size in animals is said to be the central character on which several life history traits may depend (Barbraud et al., 1999). It is often closely related to major life history traits such as fecundity, longevity, mating system and dispersal ability (Cardillo et al., 2005). Life-history traits such as dispersal ability and generation length, have been hypothesized to be important in determining species’ sensitivity to climate change and their capacity to adapt to it (Dawson et al., 2011); however, only a limited number of studies have so far provided such evidence (Poyry et al., 2009; Santini et al., 2016). In vertebrates, climate change, body size and life history traits are all inter-related in sometimes complex ways, e.g., in certain tropical lizards studied, clutch size can be mediated by body size which in turn can be affected by climate, and climate can also have size-independent effects on clutch size (Brandt & Navas, 2011). Similarly, increase in body size related to climatic warming resulted in an increase in reproductive success in a mountain population of the common lizard *Lacerta vivipara* (Chamaille-Jammes et al., 2006). However, reproductive success of mammals also declined with an increase in unfavorable habitat conditions brought about by climate warming (Isaac, 2009). Additionally, body size, activity times, fossoriality, hibernation and dispersal were shown to have mediated the response of mammals in general to climate change (Barnosky et al., 2004; Liow et al., 2009; McCain & King, 2014; Robertso et al., 2004; Sutton, Strickland & Norris, 2016; Williams & Blois, 2018).

Rodentia is characterized by species with varying life history traits and habitats, with some species showing a slow (k-selected) and some a fast (r-selected) life history strategy (Dobson & Oli, 2007) and this may facilitate or mediate their response to climate warming. Therefore, we expect these animals to show varying phenotypic response to climate warming based on both their life history traits and habitat quality.

The main aim of our study was to relate new and existing data on rodent temporal changes in body size with life history traits and environmental attributes (like habitat type, elevational range, maximum latitude and range size; from here on referred to as “habitat”) across seven families of rodents (50 species) in order to test the hypothesis that life history attributes and habitat may be an important determinant of the tendency of rodent species to respond phenotypically to climate warming and to test the applicability of the “third universal response to climate warming” (Gardner et al., 2011; Nengovhela, Baxter & Taylor, 2015) . Rodents and other small mammals (like shrews) are considered as ideal model animals because of their small size, short life spans, small dispersal distances and fast generation time as this may cause rapid morphological changes (Poroshin, Polly & Wójcik, 2010) and adaptive convergence (Samuels, 2009), which may be exacerbated by global environmental changes. Some species of rodents alter their morphology in response to extensive warming, whereas others do not (Koontz, Shepherd & Marshall, 2001).

We predicted that: (1) body size of rodents would decrease with time (year of collection), in accordance with the “third universal response to climate warming”; (2) differences in the magnitude of the temporal response would be expected between species based on differences in phylogeny, life history and habitat attributes such as mean body mass, mean litter size, r/k selection, range size, maximum latitude, fossoriality, desert adaptability, high elevational range, habitat specialization and commensality. For example, we expected r-selected and short-lived species with large litters to evolve faster and thus show more rapid evolutionary responses in body size to environmental variables compared to long-lived and k-selected species (Houle et al., 2017). Generalist and widely distributed species would be expected to adapt slower to climate change since genetic exchanges occur over wide areas, while specialist species occurring in fragmented (e.g., higher elevations) populations would be expected to respond more quickly as genetic changes can become more easily fixed. Similarly, commensal species are expected to show rapid body size increases rather than decreases due to increased food availability associated with human activity (Yom-Tov, 2003; Yom-Tov, Yom-Tov & Baagoe, 2003). Our predictions were double–tested, first for African murid rodent species (from data extracted from museum collections), and secondly by conducting a global meta-analysis of studies conducted on temporal response of rodents based on an exhaustive literature search and including the conclusions of the current study.

## Material and Methods

As described below, two approaches and datasets were analysed in the study. Firstly, mensural data of six African rodent species (from two sub-families) were collected from 739 voucher specimens in museum collections spanning about 100 years, and we used PCA and general linear models to analyse morphological trends in these data. Secondly, we added our own conclusions to a meta-database obtained from scanning all published studies where temporal trends in body or skull size were assessed. Adding the data from our recently analysed species to this global dataset, resulted in a final database of 50 species from seven rodent families. The binary response variable derived from the literature was simply the presence or absence of a reported significant temporal response of body size for each species. In the case of a significant reported change for a species, the direction (increase or decreases) was noted. We then scanned the literature (as detailed below) to obtain habitat and life history variables for each of the 50 species, which were used as predictor variables in the logistic regression model.

### Specimens sampled

#### Murid voucher museum collections

**Figure 1:**
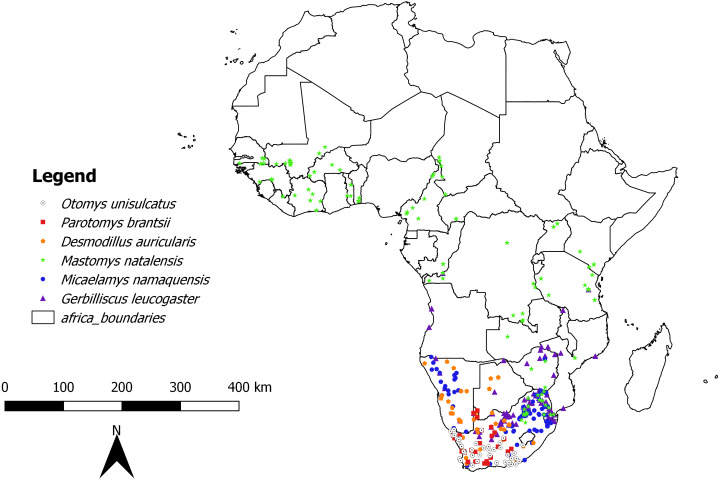
Map showing location of collecting localities of the studied species in Africa.

In the first analysis, a total of 739 skulls of rodents of six African-endemic species from two murid subfamilies, Murinae (*Otomys unisulcatus* (*n* = 102), *Parotomys brantsii* (*n* = 59), *Micaelamys namaquensis* (*n* = 142), *Mastomys natalensis* (*n* = 210)) and Gerbillinae (*Gerbilliscus leucogaster* (*n* = 132), *Desmodillus auricularis* (*n* = 94)) were measured (seeTable S1 and Fig. 1 and Appendix S1 for details of natural history parameters of the six species included). The specimens were selected from the Ditsong National Museum of Natural History (DNMNH, formerly Transvaal Museum: TM) in South Africa, Muséum National d’Histoire Naturelle, Paris, France (MNHN) and Musée Royal de l’Afrique Centrale (RMCA; Tervuren, Belgium). However, it should be kept in mind that museum collections are inherently biased both geographically and temporally (see Table S2). The species were selected based on the availability of good time series of specimens in the museum collections mentioned above. All specimens were classified into relative ages based on the degree of enamel tooth wear i.e., individuals were assigned to a relative age class of 1 (youngest) to 5 (oldest) based on tooth wear and skull shape (see Taylor & Kumirai, 2001 for Otomyini, Bates, 1985 for Gerbillini and Chimimba & Dippenaar (1994) for *Micaelamys* and *Mastomys*). Juveniles and subadults were removed and only adults (classes 3-5 for Otomyini, A-C for Gerbillini and 3-7 for *Micaelamys* and *Mastomys*) were used. The sampled percentage of specimens dating before and after 1950 was 35.32% and 64.68% respectively. In addition, we sampled the ranges of species quite broadly (Fig. 1) and there were no obvious biases in the distribution of different age groups.

### Morphology

Six cranial variables were taken to measure skull size by AN with ©TESA digital calipers to the nearest 0.01 mm: greatest length of skull (GLS), nasal width (NAW), braincase width (BW), zygomatic width (ZYW), interorbital constriction (IOC) and the maxillary tooth row length (MXTRL) (Taylor & Kumirai, 2001). We used principal component analysis (PCA) to combine the information of the six skull measurements (log transformed to standardize contribution of different variables) into a single variable for each species (PC.1, Delcros, 2012; Yom-Tov et al., 2012). This is because all of our six cranial variables were related to each other and PC.1 reflected a measure of size as indicated by the variable component loadings (Fig. S1). Therefore, the final analyses were limited to PC.1 as a proxy for cranial size hence body size. Focusing on skull size is appropriate because it has been shown to be a good indicator of overall body size in other mammals (Haigh, Stewart & Mytton, 1980) and because it has been used to evaluate temporal changes in body size in other mammals (Yom-Tov et al., 2006; Yom-Tov, Yom-Tov & Jarrell, 2008). The full raw data are presented in Table S1.

### Analysis of cranial variables for African species

All statistical analyses were conducted with R (version 3.4; R Core Team, 2017). Firstly, the existence of sexual dimorphism in size was tested using a *t*-test, with PC.1 used as an indicator of both skull and body size. None of the analyses showed significant sexual dimorphism in size, therefore the data for males and females were pooled. Both simple and multiple linear models were used to assess the relationship between PC.1 and seven predictor variables, i.e., relative age of specimens (tooth-wear class), year of collection, geographical (elevation, latitude and longitude) and environmental [mean annual temperature (Bio1) and annual precipitation (Bio12)] variables. Elevation, Bio1 and Bio12 were obtained from the Worldclim database (Hijmans et al., 2005) and ArcMap 10.4.1 was used to extract these variables for each specimen locality. Nine linear models were built in R using “Car”, “MuMIn”, and “MASS” packages for each species with seven involving individual variables, a global model that combines all the variables, and the best model determined by ranking all subsets of variables according to their Aikaike information criterion (AIC) values using the “Dredge” function of R. The model with the lowest AIC score was chosen as the most robust model. The model results showed significant effects of tooth-wear class, latitude and longitude on PC.1; to correct for these effects, the effect of temporal changes (year of collection) on PC.1 residuals obtained after multivariate linear regression on latitude, longitude and tooth-wear class was tested.

#### Meta-analysis of rodent databases

The second analysis was performed on a larger species number to test the possible effect of different life history and habitat predictors on the probability of a temporal response. We then incorporated our results of the previous analysis on six African rodent species (this study) into a meta-analysis based on published literature of temporal responses in rodents around the world, resulting in a dataset for 50 species from seven families (Table S3). The probability of temporal response was determined as a binary response variable based on metadata from literature, i.e., either a reported significant temporal response (1) or no response (0). The final group of species included in the meta-analysis included a range of small rodents such as mice (11 g) to larger rodents such as squirrels and marmots (4 kg). Studies that tested temporal changes in body size in rodents were included. In total, ten published studies were investigated (i.e., Eastman et al., 2012; Koontz, Shepherd & Marshall, 2001; Nengovhela, Baxter & Taylor, 2015; Ozgul et al., 2010; Pergams & Lawler, 2009; Pergams et al., 2015; Smith, Browning & Shepherd, 1998; Villar & Naya, 2018; Yom-Tov & Yom-Tov, 2004; Yom-Tov et al., 2012) (References in Table S3). Apart from three species in which the studied time span was seven years (*Dipodomys merriami*; Koontz, Shepherd & Marshall, 2001), eight years (*Neotoma albigula*; Smith, Browning & Shepherd, 1998) or 32 years (*Marmota flaviventris*; Ozgul et al., 2010), all of the other above studies covered a time-span of >50 years (50–106 years). These studies used different estimators of body size from skull measurements to body mass, therefore caution needs to be taken when interpreting our results. In order to determine which predictor variables may explain temporal trends in size, we quantified an array of them, and this broadly included life history traits such as mean litter size, mean body mass, and r/k selection, as well as variables related to habitat (degree of habitat specialization (generalist or specialist), maximum latitude, range size area, adaptation to fossoriality, desert adaptation, adaptation to high elevation and commensality). The information about the life history and habitat predictors was obtained from different literature, the Animal Diversity Web (ADW) website (https://animaldiversity.org), the Encyclopedia of Life (EOL) website (https://eol.org/), Happold (2013), Monadjem et al. (2015), Wilson, Lacher Jr & Mittermeier (2017) and the IUCN Redlist webpage for each species. For range size, species shape files were downloaded from the IUCN Redlist webpage for each species account and imported into QGIS using the field calculator in the Open attributes table dialog box after projecting to most relevant regional Albers Equal Areas projection. These were also used to calculate the maximum latitude and range size for each species. Range size values were log-transformed as they varied considerably by orders of magnitude. For the meta-analysis we ran generalized linear mixed models in R with the package “lme4” and rodent family used as a random factor to account partly for phylogenetic biases.

## Results

### Morphometric data analysis of African rodents

Significant differences in cranial size (PC.1) between relative age classes were documented in all six species (Table 1). As expected, in all the species, mean cranial size increased significantly from younger to older individuals (Fig. S2). In *M. namaquensis*, latitude, longitude, temperature and rainfall were all significantly correlated with PC.1 (latitude and temperature negatively, longitude and rainfall positively). In *M. natalensis*, latitude, longitude and rainfall were significantly correlated with PC.1 (latitude and rainfall positively and longitude negatively). The remaining four species (*D. auricularis*, *G. leucogaster*, *O. unisulcatus* and *P. brantsii*) showed no significant correlation with the above variables (*p* > 0.05) except for *G. leucogaster*, for which rainfall and longitude were positively correlated with PC.1 (Table 1).

**Table 1:** Akaike’s information criterion (AIC) values, coefficients of determination (*r*^2^) and degrees of freedom (df) for nine models per species fitted to explain changes in cranial size (PC.1) of six African rodent species. *Micaelamys namaquensis* (*n* = 142), *Gerbilliscus leucogaste r* (*n* = 132), *Desmodillus auricularis* (*n* = 94), *Mastomys natalensis* (*n* = 210), *Otomys unisulcatus* (*n* = 102) and *Parotomys brantsii* (*n* = 59). The best model (with the lowest AIC value) is shown in bold for each species. Climate values were downloaded from the WorldClim database using the software Arc-GIS version 10.4.1. Elevation was obtained from the GTOPO30 digital elevation model for Africa.

**Model (variables)**	**Correlation coefficient**	**Adj r**^2^	**(df)**	**AIC**
***Micaelamys namaquensis***				
1 Latitude	−0.180	0.114	(1,140)^*^	570.510
2 Longitude	0.173	0.260	(1,140)^*^	545.095
3 Year	0.003	0.005	(1,140)	588.433
4 Mean annual temperature	−0.026	0.080	(1,140)^*^	575.940
5 Elevation	−0.0003	0.002	(1,140)	587.984
6 Annual rainfall	0.004	0.250	(1,140)^*^	547.200
7 Tooth-wear class, TWCLS (as factor)		0.150	(3,138)^*^	566.955
8 Elevation + Year + Mean annual temperature + Annual rainfall +TWCLS		0.420	(7,134)^*^	516.182
**9 Global model**		**0.430**	(9,132)^*^	**515.851**
* Gerbilliscus leucogaster*				
1 Latitude	−0.019	0.004	(1,130)	549.200
2 Longitude	0.132	0.171	(1,130)^*^	523.940
3 Year	−0.010	0.011	(1,130)	547.1533
4 Mean annual temperature	−0.003	0.006	(1,130)	549.484
5 Elevation	0.0001	0.007	(1,130)	549.574
6 Annual rainfall	0.002	0.102	(1,130)^*^	534.465
7 Tooth-wear class, TWCLS		0.074	(2,129)^*^	539.560
**8 Latitude, Longitude, Annual rainfall, TWCLS**		**0.294**	(5,126)^*^	**506.555**
9 Global model		0.290	(8,123)^*^	510.751
* Desmodillus auricularis*				
1 Latitude	−0.035	0.007	(1,92)	399.540
2 Longitude	−0.033	0.007	(1,92)	399.615
3 Year	0.002	0.011	(1,92)	399.903
4 Mean annual temperature	0.001	0.011	(1,92)	399.915
5 Elevation	−0.0003	0.005	(1,92)	399.416
6 Annual rainfall	−0.002	0.009	(1,92)	398.050
**7 Tooth-wear class, TWCLS**		**0.214**	(2,91)^*^	**377.284**
8 Elevation + Year + Mean annual temperature + Annual rainfall + TWCLS		0.181	(6,87)^*^	384.870
9 Global model		0.191	(8,85)^*^	385.520
* Mastomys natalensis*				
1 Latitude	0.039	0.068	(1,208)^*^	856.835
2 Longitude	−0.040	0.130	(1,208)^*^	842.372
3 Year	0.025	0.160	(1,208)^*^	835.571
4 Mean annual temperature	0.007	0.012	(1,208)	868.907
5 Elevation	−0.0003	0.001	(1,208)	871.202
6 Annual rainfall	0.001	0.079	(1,208)^*^	854.330
7 Tooth-wear class, TWCLS		0.037	(4,205)^*^	866.483
8 Elevation + Year + Mean annual temperature + Annual rainfall + TWCLS		0.196	(8,201)^*^	832.578
**9 Global model**		**0.244**	(10,199)^*^	**821.628**
* Otomys unisulcatus*				
1 Latitude	−0.067	0.008	(1,100)	427.101
2 Longitude	0.012	0.010	(1,100)	427.273
3 Year	−0.011	0.007	(1,100)	425.622
4 Mean annual temperature	−0.001	0.010	(1,100)	427.320
5 Elevation	−0.0002	0.007	(1,100)	427.064
6 Annual rainfall	−0.0001	0.010	(1,100)	427.318
7 Tooth-wear class, TWCLS		0.555	(2,99)^*^	344.765
**8 Elevation + Year + Mean annual temperature + Annual rainfall + TWCLS**		**0.580**	(6,95)^*^	**343.477**
9 Global model		0.571	(8,93)^*^	346.624
* Parotomys brantsii*				
1 Latitude	0.073	0.005	(1,57)	236.604
2 Longitude	0.037	0.015	(1,57)	237.213
3 Year	0.015	0.006	(1,57)	235.964
4 Mean annual temperature	−0.003	0.017	(1,57)	237.288
5 Elevation	0.0003	0.012	(1,57)	237.040
6 Annual rainfall	0.002	0.010	(1,57)	236.910
7 Tooth-wear class, TWCLS		0.472	(2,56)^*^	199.555
**8 Elevation + Year + Mean annual temperature + Annual rainfall + TWCLS**		**0.508**	(6,52)^*^	**199.064**
9 Global model		0.509	(8,50)^*^	200.619

**Notes.**

* Denotes *p* < 0.05.

**Figure 2:**
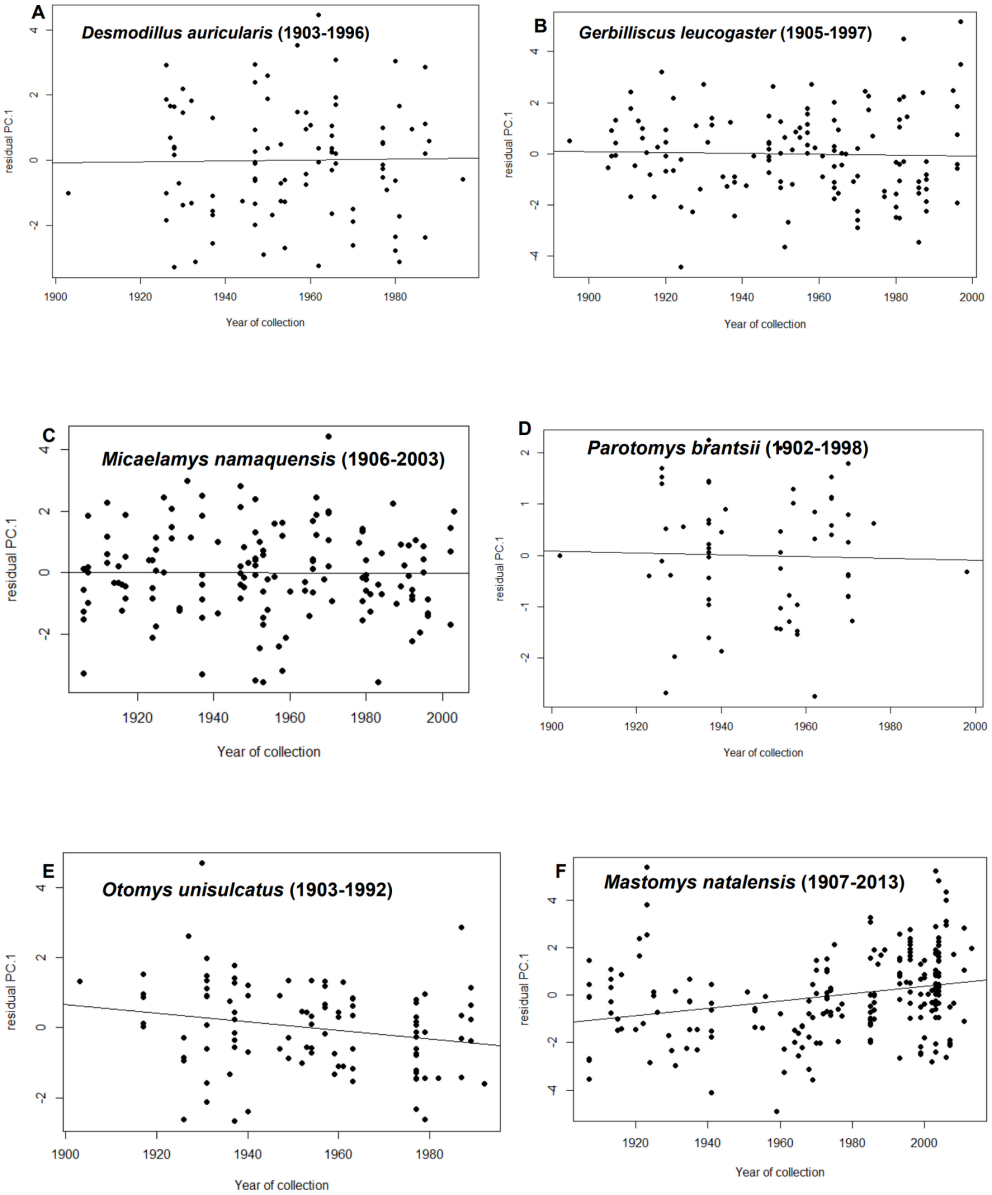
Relationship between residuals of PC.1 (skull size) and year of collection for each species included in the analysis. (A) *Desmodillus auricularis* (1903–1996), (B) *Gerbilliscus leucogaster* (1905–1997), (C) *Micaelamys namaquensis* (1907–2013), (D) *Parotomys brantsii* (1902–1998), (E) *Otomys unisulcatus* (1903–1992), (F) *Mastomys natalensis* (1907-2013)

After statistically correcting for tooth-wear class, latitude, longitude and elevation, residuals from only two species showed temporal trends i.e., *M. natalensis* and *O. unisulcatus*. In *M. natalensis*, year of collection was significantly positively correlated with PC.1 (r^2^_adj_ = 0.07, *p* < 0.0001) and in *O. unisulcatus*, year of collection was marginally significantly negatively correlated with PC.1 (r^2^_adj_ = 0.04, *p* = 0.032) (Fig. 2).

### Meta-analysis of combined database

Among 50 rodent species reviewed, 29 showed no significant change, eight showed a significant increase in size and 13 showed a decline in size (see Table S3). Out of the seven families, three had no species that varied temporally, Dipodidae (two species), kangaroo rats Heteromyidae (two species) and pocket gophers Geomidae (two species). A “mole-rat” (Spalacidae, one species) showed a temporal response (increase), while around half the species of murids, cricetids and sciurids (squirrels) showed a temporal response. However, the direction of changes varied between families. In Muridae (*n* = 18), both increases (four) and decreases (four) were recorded, while in Cricetidae (*n* = 18), only decreases (nine) were observed, and in Sciuridae (*n* = 7), only increases (three) were observed. Using a binomial logistic regression model, we found that none of the several tested predictors (i.e., maximum latitude, range size area, fossoriality, high- elevation, habitat specialist, commensal, desert adapted, mean body mass, r/k selection and mean litter size) could significantly explain the probability of a rodent species to show a temporal effect on body size (Table 2).

**Table 2:** Generalized linear mixed model results for three life history traits (underlined in table below) and seven habitat variables (10 predictors in total) used to explain variation in probability of temporal response in 50 rodents species. Rodent family was taken as a random factor to account partly for phylogenetic biases. The four quantitative variables were litter size, body mass, maximum latitude and range size area. For the six binary variables, default values were “1”, i.e. fossoriality, habitat specialist, commensal, dessert-dwelling, high elevation, r- or k-selected (r=1, k=0). Estimates represent comparisons with state “0”, e.g., non-fossorial rodents (“0”) have lower probability (negative sign of estimate) of temporal change than fossorial (“1”).

	**Estimate**	**Std. Error**	**z value**	**Pr(>—z—)**
(Intercept)	−6.16531	4.52460	−1.363	0.173
Mean.litter.size	0.03711	0.29563	0.126	0.900
log(Mean.body.mass..g.)	0.85106	0.62155	1.369	0.171
as.factor(Fossoriality)1	−0.09800	0.85207	−0.115	0.908
as.factor(Habitat)1	1.40864	0.97997	1.437	0.151
as.factor(Commensal)1	0.28841	1.16262	0.248	0.804
as.factor(Desert)1	0.12712	0.88419	0.144	0.886
as.factor(Elevation)1	1.48609	0.83743	1.775	0.076
as.factor(r.k)1	1.07638	1.21217	0.888	0.375
maximum.latitude	0.02137	0.02530	0.845	0.398
log(range.size.Area)	−0.05335	0.21863	−0.244	0.807

## Discussion

### Temporal size trends in African rodents

Few data are available on temporal trends in body size in African rodents. Nengovhela, Baxter & Taylor (2015) assessed temporal changes in skull size over 100 years in museum collections of two South African rodent species of the murine Tribe Otomyini (laminate-toothed rats), *Otomys auratus* and *O. angoniensis*, revealing a significant decrease in size in both species. The decrease in size over time was greater (6%) in the former, higher-elevation and grassland-associated species compared to the latter, savanna-associated species (3% decrease). It was argued that the narrower-niche habitat and more restricted high-elevation range of *O. auratus* compared to *O. angoniensis* may explain this difference (Nengovhela, Baxter & Taylor, 2015). To better understand the generality and possible life history and habitat causes of temporal size changes in African rodents, we here added comparative novel data from museum collections of a further six species of rodents, two from the murine Tribe Otomyini (*O. unisulcatus* from semi-arid habitats and *Parotomys brantsi* from desert habitats), two additional and widespread murine species from the savanna biome (*Mastomys natalensis* and *Micaelamys namaquensis*) and two gerbils (Subfamily Gerbillinae) occurring in dry savannas (*Gerbilliscus leucogaster*) and true deserts (*Desmodillus auricularis*). We only found significant temporal trends in *M. natalensis* (increase) and *O. unisulcatus* (decrease). Taken together with the data of Nengovhela, Baxter & Taylor (2015), this means that only four out of eight African rodent species surveyed show a significant temporal effect on skull size over a 100 years period. The direction of change was not uniform with three species showing decreases and one an increase. This question both the universality and causality of the so-called third universal response to climate change (Gardner et al., 2011). Apart from the possibility of increased temperature explaining declines in body size, the increase in body size in *M. natalensis*, which is a widespread agricultural pest in Africa, may be better explained by nutrition (large increases in rural agriculture and settlements in Africa over the past 100 years). As discussed below, these data from African rodents were appended to a global meta-database which showed similar trends.

### Life history and habitat do not seem to predict response to climate warming

Rodents have been said to display a very wide variation of life history patterns (Samuels, 2009) with body size reported to be related to a multitude of life history traits (e.g., gestation length, reproductive rate, home range size, basal metabolic rate, etc, (Webster, Gittleman & Purvis, 2004). Based on our generalized linear mixed model of our global meta-database of 50 rodent species (including also our current data), we found that none of our chosen predictors could significantly explain the probability of a temporal response. However, looking at each predictor, elevation even though insignificant, showed a 60% chance of temporal changes in high altitude species compared to 30% in low altitude species. This might be due to the fact that high elevation habitats are more prone to fragmentation (Naya, Naya & Cook, 2017), causing rapid morphological changes. This may be equated to the rapid morphological changes shown on islands (see Pergams & Ashley, 1999; Pergams et al., 2015). Villar & Naya (2018) showed that rodent species displaying life history traits like torpor or hibernation (heterothermic species) responded less to climate change over time than did homeothermic species. This indicates that other life history attributes may also explain the probability (and direction) of temporal responses in rodents. The non-significant results in our chosen predictors may be caused by the small sample size, effects of phylogeny, unequal sampling of different rodent families and the subjectivity of scoring some of the predictors. Our sample was based on only 50 species out of a total of 2565 (1.95%) currently recognized rodent species.

### Body size response to climate change is not so universal in rodents

Both in the new data added for six African rodents (where one species increased in size, one decreased and four did not change), and for our global dataset of 50 species, we found variable responses to general temporal changes over an approximately 100 year period, with increases, decreases and no change. These differing temporal responses in rodents have been reported in other studies, for example, Villar & Naya (2018) showed a temporal decrease in body size of about seven rodent species and no response in ten species. A similar trend was reported in Eastman et al. (2012), Nengovhela, Baxter & Taylor (2015), Ozgul et al. (2010), Pergams & Lawler (2009), with either decreases, increases and no changes reported (Table S3). Changes in body size have been attributed to differing factors such as global warming, increased food availability, snow melt, longer growing season (Eastman et al., 2012; Nengovhela, Baxter & Taylor, 2015; Pergams & Lawler, 2009; Smith, Browning & Shepherd, 1998; Villar & Naya, 2018; Yom-Tov & Yom-Tov, 2004; Yom-Tov et al., 2012) with some of these studies providing support for Bergmann’s clines. As clarified by Blackburn, Gaston & Loder (1999), Bergmann’s Rule was originally applied to closely related species, but as noted by James (1970) it was later adapted to apply to ‘races of a species’ (see Mayr, 1956). Blackburn, Gaston & Loder (1999) proposed restricting the definition of Bergmann’s Rule to cases of interspecific patterns and using the term ‘James’s Rule’ to apply to cases of intraspecific variation. At the intraspecific level (James’s Rule sensu Blackburn, Gaston & Loder, 1999), if the temporal decrease in cranial size of *O. unisulcatus* is due to temperature change, and the underlying mechanism is Bergmann’s Rule, we would expect that the observed latitudinal trends should also be correlated with temperature. However, our model for *O. unisulcatus* showed no correlation of cranial size with temperature. Interestingly, our model for *M. natalensis* showed positive correlation of precipitation with cranial size, coupled with the temporal increase in body size. This conforms to the productivity hypothesis or what McNab (2010) refers to as Resource Rule, which posits that “mammalian species will become larger or smaller depending on the size, abundance and availability of resources”. Our results are consistent with what have been reported in Alhajeri & Steppan (2016), who showed that increased body mass was related to increasing precipitation variables (hence, increased food availability) supporting James (1970) and Yom-Tov & Geffen (2006). However, the relationship between precipitation and primary productivity is not as straight forward as other factors such as temperature, also plays a role in determining primary productivity (Yom-Tov & Geffen, 2006; Yom-Tov & Geffen, 2011). Alhajeri, Porto & Maestri (2019) found no support of resource availability playing a role in explaining geographic variation in rodent body size.

### Body size response to climate warming in other terrestrial vertebrates

A variety of phenotypic responses to climate change, including increases, decreases and no changes have been reported in different vertebrate taxa including Passeriformes (Gardner, Heinsohn & Joseph, 2009; Gardner et al., 2014; Goodman et al., 2012; Husby, Hille & Visser, 2011; McCoy, 2012; Moreno-Rueda & Rivas, 2007; Yom-Tov, 2001), Carnivora (Meiri et al., 2009; Yom-Tov, Yom-Tov & Baagoe, 2003; Yom-Tov et al., 2006; Yom-Tov, Yom-Tov & Jarrell, 2008; Yom-Tov et al., 2010b), *Ovis aries* (Ozgul et al., 2009), *Chroicocephalus scopulinus* (Teplitsky et al., 2008) and *Sorex cinereus* (Yom-Tov & Yom-Tov, 2005). Factors invoked to explain the diversity of responses included *inter alia*, temperature, climate variability, increased productivity, shorter and milder winters, stress and diet.

### Difficulties in measuring body size & other study limitations

In this study, it is of importance to acknowledge potential drawbacks, consider other alternative explanations for the lack of temporal responses in our study groups, and to interpret our results with caution. This study has failed to incorporate other life history and factors that may play a role in explaining the body size temporal response of certain species to warming. Data on some life history variables (like life span, feeding habits, fecundity) are missing although we know they vary in general between species e.g., life span, small murids in general have shorter life spans than for example mole-rats and squirrels.

Other possible reasons for the widely divergent results concerning temporal changes in body size of rodents could be due to how it is measured. Different studies have used different proxies as a measure of body size i.e., body mass, skull length, cranial size and body length (Alhajeri & Steppan, 2016; Nengovhela, Baxter & Taylor, 2015; Pergams & Lawler, 2009; Villar & Naya, 2018; Yom-Tov & Yom-Tov, 2004)...... Mori et al. (2019) showed skull size to be positively correlated with body size across the *Hystrix* species and confirmed skull size to be a suitable proxy for body size. Similarly, Eastman et al. (2012) regarded skull length as a trait positively correlated with body size. Interestingly, although all these studies have used different measurements as a proxy for body size, they have all indicated a reduction, an increase and a no change response.

## Conclusions

In conclusion, we found that the “third universal response to warming” (Gardner et al., 2011) is not as universal as claimed and that trends are conflicting and not explained by any of the life history or habitat factors we considered. We understand there are other factors that may play a role in explaining the temporal response or lack thereof in this study. In our study, only one species (*O. unisulcatus*) showed a temporal decrease in body size. Additionally, causes driving body size trends are still not clear as our data are correlational rather than causal. Future studies should also test our predictors from large samples of species within a single family and adding predictors such as gestation length, reproductive rate, metabolic rate, neonatal weight, heterothermy versus homoethermy and population density. A limitation of this study is that we assume that the body size changes are genetic, the same assumption made in most of the papers reviewed here. However, we do understand that sometimes body changes may reflect a non-genetic phenotypic response (Khokhlova et al., 2001).

##  Supplemental Information

10.7717/peerj.9792/supp-1 Supplemental Information 1 Raw data of the studied specimens for each species investigated

10.7717/peerj.9792/supp-2 Supplemental Information 2 Data distribution of each species based on age class and year intervals Fossorial: Species that dig burrows
Desert: Species whose habitat included desert were considered desert species.
High-elevation: Species whose elevational range exceeds 2500 m were considered as high elevation.
Habitat specialist: Species who occur in one/two habitats were considered specialist as compared to those species whose habitat includes more than two.
Commensal: Species found in settlements and those considered pests (agricultural areas)
Predictors: Likely factors that could drive the temporal size trend and cited in the reference.

10.7717/peerj.9792/supp-3 Supplemental Information 3 Studies investigating temporal trends in relation to climate warming and Bergmann’s Rule for rodents with each species life history parameters and environmental attributes Fossorial: Species that dig burrows
Desert: Species whose habitat included desert were considered desert species.
High-elevation: Species whose elevational range exceeds 2500 m were considered as high elevation.
Habitat specialist: Species who occur in one/two habitats were considered specialist as compared to those species whose habitat includes more than two.
Commensal: Species found in settlements and those considered pests (agricultural areas)
Predictors: Likely factors that could drive the temporal size trend and cited in the reference.

10.7717/peerj.9792/supp-4 Supplemental Information 4 Plot of component scores of the six species The associated variable loadings of the first two Principal Components (PC 1: PC 2): GLS (-0.47:0.01), MXTRL (-0.29:-0.67), NAW (-0.38:-0.47), IOC (-0.38:0.46), ZYW (-0.47:0.14), BW (-0.43:0.30).

10.7717/peerj.9792/supp-5 Supplemental Information 5 Differences in cranial size (PC.1) by age class category per species

10.7717/peerj.9792/supp-6 Supplemental Information 6 Natural history of African species studied

## Additional Information and Declarations

### Data Availability

The following information was supplied regarding data availability: The raw cranial measurements (six) are available in Table S1. The six cranial measurements were used for statistical analysis to calculate the overall skull size of each species. The 739 specimens are available from the Ditsong National Museum of Natural History (DNMNH), Royal Museum for Central Africa, Tervuren (RMCA) and Museum National d’Histoire Naturelle (MNHN). In addition to the cranial measurements, other raw variables used in this study such as the specimen identifiers are also available in Table S1.
