# Supplementary material for: Life history and habitat do not mediate temporal changes in body size due to climate warming in rodents
Source: PeerJ. 2020 Sep 24;8:e9792. doi: 10.7717/peerj.9792 (PMC7520088; doi:10.7717/peerj.9792)
Supplement: Supplemental Information 2 — Fossorial: Species that dig burrows Desert: Species whose habitat included desert were considered desert species. High-elevation: Species whose elevational range exceeds 2500 m were considered as high elevation. Habitat specialist: Species who occur in one/two habitats were considered specialist as compared to those species whose habitat includes more than two. Commensal: Species found in settlements and those considered pests (agricultural areas) Predictors: Likely factors that could drive the temporal size trend and cited in the reference. [file peerj-08-9792-s002.docx]

| **Species Name** | **Age Class** | | | | | | | **Year Interval** | | |
| --- | --- | --- | --- | --- | --- | --- | --- | --- | --- | --- |
|  | **1** | **2** | **3** | **4** | **5** | **6** | **7** | **1900-1950** | **1950-2000** | **2000-2013** |
| *Otomys unisulcatus* | - | - | 6 | 44 | 52 | - | - | 43 | 59 | - |
| *Parotomys brantsii* | - | - | 3 | 24 | 32 | - | - | 27 | 32 | - |
| *Gerbilliscus lecougaster* | 59 | 71 | 2 | - | - | - | - | 51 | 81 | - |
| *Desmodillus auricularis* | 13 | 52 | 29 | - | - | - | - | 36 | 58 | - |
| *Mastomys natalensis* | - | - | 3 | 54 | 66 | 50 | 37 | 44 | 101 | 65 |
| *Micaelamys namaquensis* | - | - | - | 75 | 43 | 17 | 7 | 60 | 78 | 4 |
