# Supplementary material for: Life history and habitat do not mediate temporal changes in body size due to climate warming in rodents
Source: PeerJ. 2020 Sep 24;8:e9792. doi: 10.7717/peerj.9792 (PMC7520088; doi:10.7717/peerj.9792)
Supplement: Supplemental Information 3 — Fossorial: Species that dig burrows Desert: Species whose habitat included desert were considered desert species. High-elevation: Species whose elevational range exceeds 2500 m were considered as high elevation. Habitat specialist: Species who occur in one/two habitats were considered specialist as compared to those species whose habitat includes more than two. Commensal: Species found in settlements and those considered pests (agricultural areas) Predictors: Likely factors that could drive the temporal size trend and cited in the reference. [file peerj-08-9792-s003.docx]

| **Family** | **Genus** | **Species** | **Continent/ Hemisphere** | **Maximum latitude** | **Range size area** | **Mean litter size** | **Mean body mass (g)** | **Fossorial/ not** | **Desert/not** | **high- elevation/ not** | **Habitat specialist/ not** | **Commensal/not** | **Temporal size trend** | **Predictors** | **Time span** | **Reference** |
| --- | --- | --- | --- | --- | --- | --- | --- | --- | --- | --- | --- | --- | --- | --- | --- | --- |
| Cricetidae | *Abrothrix* | *longipilis* | SA | 30.29 | 717952.11  5 km2 | 3.85 | 36.7 | Yes | No | No | No | No | Decrease | Increased temperature | 100+ years | Pergams & Lawler 2009 |
| Cricetidae | *Abrothrix* | *olivaceus* | SA | 18.39 | 1085022  km2 | 3.85 | 39 | - | No | No | No | No | No response | - | 100+ years | Pergams & Lawler 2009 |
| Cricetidae | *Abrothrix* | *sanborni* | SA | 42.58 | 4530.491  km2 | 3.85 | 25 | No | No | No | Yes | No | No response | - | 100+ years | Pergams & Lawler 2009 |
| Cricetidae | *Dicrostonyx* | *groenlandicus* | NA | 83.63 | 2561987  km2 | 3.4 | 66 | Yes | No | No | Yes | No | No response | - | 1956-2015 | Villar & Naya, 2018 |
| Cricetidae | *Eothenomys* | *smithii* | Asia | 38.43963 | 145776.93 km2 | 4.4 | 42.5 | No | No | No | No | No | No response |  | 1920-1989 | Yom-Tov & Yom-Tov 2004 |
| Cricetidae | *Lemmus* | *trimucronatus* | NA | 74.56 | 5779510  km2 | 3.7 | 80 | No | No | No | Yes | No | Decrease | Global warming | 1951-2015 | Villar & Naya, 2018 |
| Cricetidae | Microtus | agrestis | Mediterranea, Europe | 71.1225 | 20524261.42 km2 | 4.4 | 42.5 | No | No | No | No | No | No response |  | 1895-2004 | Yom-Tov et al 2012 |
| Cricetidae | *Microtus* | *longicaudus* | NA | 68.14 | 40214210  km2 | 5 | 37 | Yes | No | Yes | No | Yes | No response | - | 1956-2015 | Villar & Naya, 2018 |
| Cricetidae | *Microtus* | *mexicanus* | NA | 37.58 | 854502.16  km2 | 2.3 | 35 | No | No | Yes | Yes | No | No response | - | 100+ years | Pergams & Lawler 2009 |
| Cricetidae | *Microtus* | *miurus* | NA | 70.89 | 1431571  km2 | 8.2 | 41 | Yes | No | Yes | Yes | No | Decrease | Global warming | 1947-2015 | Villar & Naya, 2018 |
| Cricetidae | *Microtus* | *oeconomus* | Europe, Asia, NA | 75.54 | 19507729  km2 | 6.9 | 50 | No | No | Yes | Yes | No | Decrease | Global warming | 1947-2015 | Villar & Naya, 2018 |
| Cricetidae | *Microtus* | *pennsylvanicus* | NA | 70.25 | 11963797  km2 | 5.5 | 44 | Yes | No | Unknown | No | Yes | Decrease | Global warming | 1928-2015 | Villar & Naya, 2018 |
| Cricetidae | *Myodes* | *gapperi* | NA | 62.43 | 8370580  km2 | 3.7 | 21 | Yes | No | Yes | Yes | No | Decrease | Global warming | 1932-2015 | Villar & Naya, 2018 |
| Cricetidae | *Neotoma* | *albigula* | NA | 39.12 | 845054  km2 | 2 | 197 | No | Yes | No | No | Yes | Decrease | Climate warming | 8 years | Smith et al. 1998 |
| Cricetidae | *Peromyscus* | *leucopus* | NA | 51.13 | 5831155  km2 | 4.5 | 23 | No | Yes | No | No | No | Decrease | Global warming | 1929-2015 | Villar & Naya, 2018 |
| Cricetidae | *Peromyscus* | *maniculatus* | NA | 65.65 | 13316662  km2 | 5 | 20 | No | Yes | Yes | No | No | Decrease | Global warming | 1932-2015 | Villar & Naya, 2018 |
| Cricetidae | *Reithrodontomys* | *megalotis* | NA | 50.61 | 5116675  km2 | 4 | 11 | No | Yes | Yes | No | No | No response |  | 1956-2016 | Villar & Naya, 2018 |
| Cricetidae | *Sigmodon* | *hispidus* | NA | 40.94 | 2615709  km2 | 5 | 159 | No | Yes | No | No | Yes | No response |  | 1933-2015 | Villar & Naya, 2018 |
| Dipodidae | *Napaeozapus* | *insignis* | NA | 54.23 | 2009949  km2 | 4.5 | 25 | No | No | No | Yes | No | No response |  | 1937-2013 | Villar & Naya, 2018 |
| Dipodidae | *Zapus* | *hudsonius* | NA | 65.03 | 8737705  km2 | 5 | 18 | No | No | No | No | Yes | No response |  | 1933-2015 | Villar & Naya, 2018 |
| Geomyidae | *Thomomys* | *bottae* | NA | 42.96 | 1541296  km2 | 5.6 | 116 | Yes | Yes | Yes | No | No | No response |  | 1956-2016 | Villar & Naya, 2018 |
| Geomyidae | *Thomomys* | *umbrinus* | NA | 32.86 | 469421.35  9 km2 | 4 | 130 | Yes | Yes | Yes | No | No | No response |  | 100+ years | Pergams & Lawler 2009 |
| Heteromyidae | *Chaetodipus* | *fallax* | NA | 34.36 | 46906.768  km2 | 3 | 20 | Yes | Yes | No | No | No | No response |  | 100+year | Pergams & Lawler 2009 |
| Heteromyidae | *Dipodomys* | *merriami* | NA | 40.39 | 1423668  km2 | 3 | 42 | Yes | Yes | No | No | No | No response |  | 1989-1996 | Koontz et al.  2001 |
| Muridae | *Apodemus* | *flavicollis* | Mediterranea, Europe | 64.03066 | 9591684.17 km2 | 5.5 | 31.6 | Yes | No | No | No | Yes | No response |  | 1895-2002 | Yom-Tov et al 2012 |
| Muridae | *Apodemus* | *speciosus* | Asia | 45.52314 | 467783.451 km2 | 5 | 43.75 | No | No | No | No | Yes | Increase | Warmer autumn, summer and winter | 1949-1989 | Yom-Tov & Yom-Tov 2004 |
| Muridae | Apodemus | sylvaticus | Mediterranea, Europe | 66.53463 | 7555528.104 km2 | 5.2 | 39.4 | Yes | No | Yes | No | Yes | No response |  | 1847-2002 | Yom-Tov et al 2012 |
| Muridae | *Desmodillus* | *auricularis* | Africa | 12.58 | 1955062  km2 | 3 | 46 | Yes | Yes | No | Yes | No | No response |  | 1903-1996 | Current study |
| Muridae | *Gerbilliscus* | *leucogaster* | Africa | 4.92 | 57652391  0 km2 | 5 | 72.2 | No | No | No | Yes | Yes | No response |  | 1905-1997 | Current study |
| Muridae | *Lophuromys* | *flavopunctatus* | Africa | 9.95 | 1820851.7  4 km2 | 2.4 | 69 | No | No | Yes | Yes | No | Decrease | Increased temperature | 100+ years | Pergams & Lawler 2009 |
| Muridae | *Mastomys* | *natalensis (sl)* | Africa | 16.91 | 16655238  km2 | 6.5 | 41 | No | No | No | No | Yes | Increase | Global warming (rainfall) | 1907-2013 | Current study |
| Muridae | *Micaelamys* | *namaquensis* | Africa | 14.85 | 3338607  km2 | 3.3 | 48 | No | Yes | No | No | Yes | No response |  | 1906-2003 | Current study |
| Muridae | *Mus* | *musculus* | Cosmopoli tan | 73.53 | 15150382  5 km2 | 5 | 21 | No | No | No | No | Yes | No response |  | 1930-2015 | Villar & Naya, 2018 |
| Muridae | *Oligoryzomys* | *longicaudatus* | SA | 25.90 | 629012.30  4 km2 | 5 | 28 | No | No | unknown | No | No | Increase | Increased temperature | 100+ years | Pergams & Lawler 2009 |
| Muridae | *Otomys* | *angoniensis* | Africa | 1.02 | 595014  km2 | 3.1 | 114.3 | No | No | No | Yes | No | Decrease | Global warming | 1906-2013 | Nengovhela et al. 2015 |
| Muridae | *Otomys* | *auratus* | Africa | 18.04 | 519018  km2 | 2.8 | 127.3 | No | No | Yes | Yes | No | Decrease | Global warming | 1906-2013 | Nengovhela et al. 2015 |
| Muridae | *Otomys* | *unisulcatus* | Africa | 27.43 | 374311  km2 | 2.09 | 124.5 | No | Yes | No | Yes | No | Decrease | Global warming | 1903-1992 | Current study |
| Muridae | *Parotomys* | *brantsii* | Africa | 23.44 | 679441  km2 | 3.4 | 95 | Yes | Yes | No | Yes | No | No response |  | 1902-1998 | Current study |
| Muridae | *Phyllotis* | *xanthopygus* | SA | 10.68 | 1499353.4  12 km2 | 4.7 | 55 | No | No | Yes | No | No | No response |  | 100+ years | Pergams & Lawler 2009 |
| Muridae | *Praomys* | *jacksoni* | Africa | 11.11 | 3744033.0  37 km2 | 3.8 | 41 | No | No | Yes | No | No | No response |  | 100+ years | Pergams & Lawler 2009 |
| Muridae | *Rattus* | *rattus* | Cosmopoli tan | 61.72 | 43666656  km2 | 8 | 200 | No | No | No | No | Yes | Increase | Island introduction | 1940-2000 | Pergams et al.  2015 |
| Muridae | *Rattus* | *tanezumi* | Asia | 45.61 | 9717373.2  42 km2 | 8 | 140 | No | No | No | No | Yes | No response |  | 100+ years | Pergams & Lawler 2009 |
| Sciuridae | *Callospermophilus* | *lateralis* | NA | 56.39 | 1704569  km2 | 5 | 158 | Yes | No | Yes | No | Yes | Increase | Temperature & snow melt | 1902-1950; 2000-2008 | Eastman et al.  2012 |
| Sciuridae | *Marmota* | *flaviventris* | NA | 51.13 | 1718209  km2 | 4 | 3350 | Yes | No | Yes | Yes | No | Increase | Longer growing season | 1976-2008 | Ozgul et al.  2010 |
| Sciuridae | *Otospermophilus* | *beecheyi* | NA | 46.93 | 467249  km2 | 5 | 500 | Yes | No | Yes | No | Yes | No response |  | 1902–1950;2000–2008 | Eastman et al.  2012 |
| Sciuridae | *Sciurus* | *carolinensis* | NA | 53.41 | 4083837.5  59 km2 | 3 | 540.33 | No | No | No | No | Yes | No response |  | 100+ years | Pergams & Lawler 2009 |
| Sciuridae | *Tamias* | *striatus* | NA | 51.67 | 4197494  km2 | 4 | 96 | Yes | No | No | No | No | No response |  | 100+ years; 1925-2015 | Pergams & Lawler 2009; Villar & Naya, 2018 |
| Sciuridae | *Tamiasciurus* | *hudsonicus* | NA | 67.78 | 10363614  km2 | 4 | 213 | No | No | No | No | Yes | No response |  | 1931-2016 | Villar & Naya, 2018 |
| Sciuridae | *Urocitellus* | *beldingi* | NA | 45.91 | 337683  km2 | 5.7 | 290 | Yes | No | Yes | No | Yes | Increase | Temperature & snow melt | 1902-1950; 2000-2008 | Eastman et al.  2012 |
| Spalacidae | *Tachyoryctes* | *splendens* | Africa | 14.59 | 680054.69  2 km2 | 1.65 | 265.7 | Yes | No | Yes | No | Yes | Increase | Increased temperature | 100+ years | Pergams & Lawler 2009 |

**References in Table:**

Eastman LM, Morelli TL, Rowe KC, Conroy CJ, Moritz C. 2012. Size increase in high elevation ground squirrels over the last century. Global Change Biology 18(5): 1499–1508 DOI 10.1111/j.1365-2486.2012.02644.x.

Koontz TL, Shepherd UL, Marshall D. 2001. The effects of climate change on Merriam’s kangaroo rat, *Dipodomys merriami*. Journal of Arid Environments 49(3): 581-591 DOI 10.1006/jare.2001.0806.

Nengovhela A, Baxter RM, Taylor PJ. 2015. Temporal changes in cranial size in South African vlei rats (*Otomys*): evidence for the “third universal response to warming.” African Zoology 50(3): 233–239 DOI 10.1080/15627020.2015.1052014.

Ozgul A, Childs DZ, Oli MK, Armitage KB, Blumstein DT, Olson LE, Tuljapurkar S, Coulson T. 2010. Coupled dynamics of body mass and population growth in response to environmental change. Nature 466(7305): 482–485 DOI 10.1038/nature09210.

Pergams ORW, Byrn D, Lee KLY, Jackson R. 2015. Rapid morphological change in black rats (*Rattus rattus*) after an island introduction. PeerJ 3: e812 DOI 10.7717/peerj.812.

Pergams ORW, Lawler JJ. 2009. Recent and widespread rapid morphological change in rodents. PLOS ONE 4(7): e6452.

Smith FA, Browning H, Shepherd UL. 1998. The influence of climate change on the body mass of woodrats *Neotoma* in an arid region of New Mexico, USA. Ecography 21(2): 140–148 DOI 10.1111/j.1600-0587.1998.tb00668.x.

Villar CH, Naya DE. 2018. Climate change and temporal trends in body size: the case of rodents. Oikos 127(8): 1186–1194 DOI 10.1111/oik.04884.

Yom-Tov Y, Yom-Tov S. 2004. Climatic change and body size in two species of Japanese rodents. Biological Journal of the Linnean Society 82(2): 263–267 DOI 10.1111/j.1095-8312.2004.00357.x.

Yom-Tov Y, Yom-Tov S, Jensen TS, Baagoe H. 2012. No recent temporal changes in body size of three Danish rodents. Acta Theriologica 57(1): 59–63 DOI 10.1007/s13364-011-0052-y.
