# Supplementary figures and images for: Life history and habitat do not mediate temporal changes in body size due to climate warming in rodents
Source: PeerJ. 2020 Sep 24;8:e9792. doi: 10.7717/peerj.9792 (PMC7520088; doi:10.7717/peerj.9792)

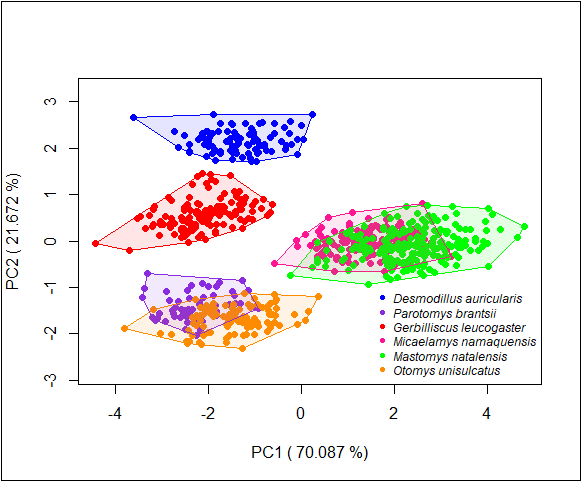

Supplement: Supplemental Information 4 — The associated variable loadings of the first two Principal Components (PC 1: PC 2): GLS (-0.47:0.01), MXTRL (-0.29:-0.67), NAW (-0.38:-0.47), IOC (-0.38:0.46), ZYW (-0.47:0.14), BW (-0.43:0.30). [file peerj-08-9792-s004.png]

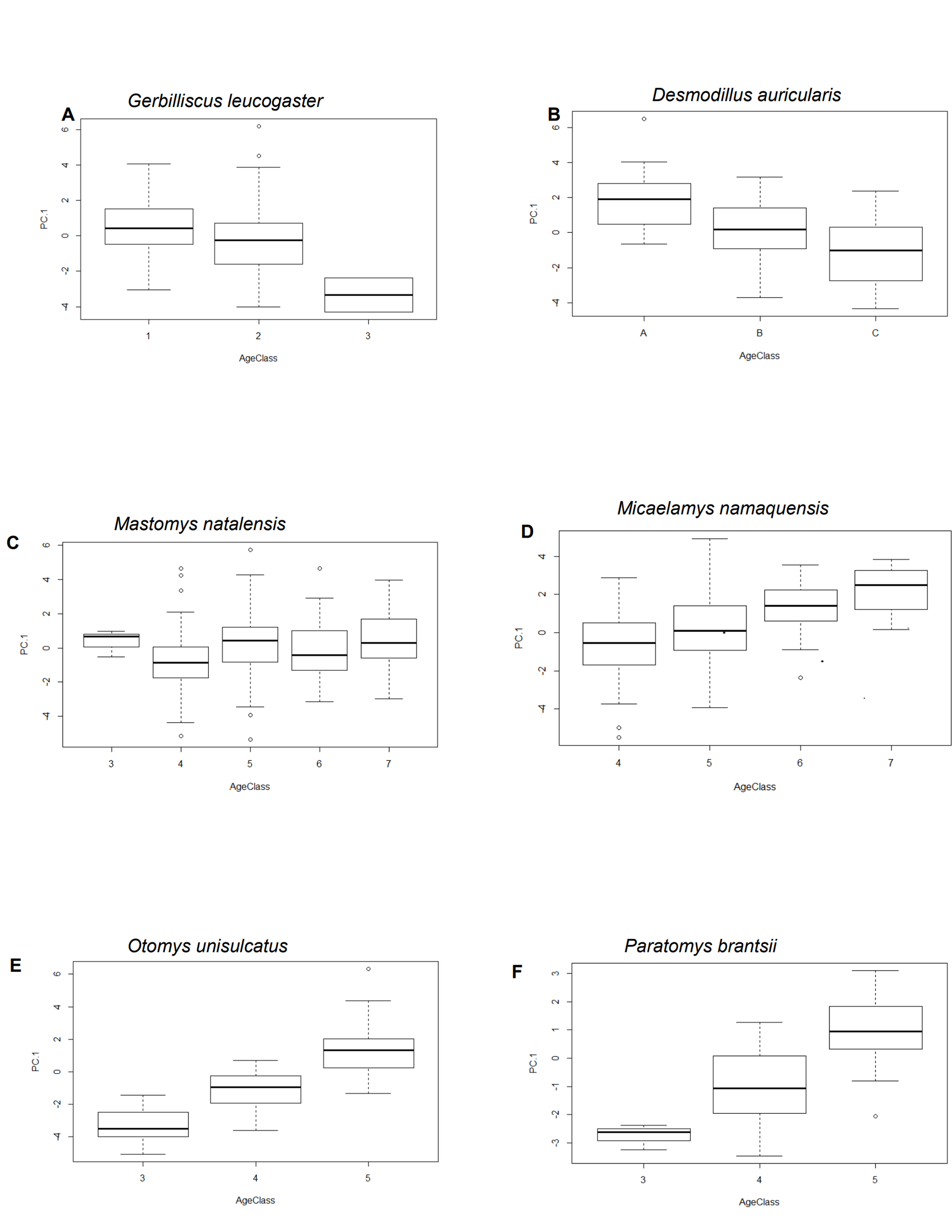

Supplement: Supplemental Information 5 [file peerj-08-9792-s005.png]
