# Supplementary material for: Life history and habitat do not mediate temporal changes in body size due to climate warming in rodents
Source: PeerJ. 2020 Sep 24;8:e9792. doi: 10.7717/peerj.9792 (PMC7520088; doi:10.7717/peerj.9792)
Supplement: Supplemental Information 6 [file peerj-08-9792-s006.docx]

First, we used museum collections hosting long time series to investigate the morphological response of six species of African Muridae rodents to global climate warming, so as to build a new dataset for this continent, and compare it with the global literature. The African species were chosen because Nengovhela, Baxter & Taylor (2015) is the only African study known by the authors to investigate rodent morphology and climate warming to date. Among the selected species, we chose Murinae and Gerbillinae, which have different ecological requirements as well as life history parameters but have similar body sizes ranges (gerbils 46-72, murines 41-95g). The bushveld gerbil Gerbilliscus leucogaster is a medium-sized rodent that is endemic to Africa, widely distributed and belongs to the tribe Taterillini. It is nocturnal and it inhabits open grasslands and wooded savannas on light sandy and soft soils. Its diet consists of insects, seeds and herbage. It has short gestation length of 28 days, large litter-size, a life span of about 12 months and it is considered to have a social structure. Desmodillus auricularis also belongs to the tribe Taterillini, is endemic to Africa and widely distributed in the arid savanna and desert zone of southwestern Africa. A desert species that prefers hard or consolidated soils with low shrubs or sparse grasslands, it is nocturnal and omnivorous consuming seeds, insects and green leaves. It is asocial and solitary. Its reproduction is throughout the year with a gestation length of 21 days, a life span of aboit 1-2 years and a mean litter size of three young. Parotomys brantsii and Otomys unisulcatus are whistling rats and bush Karoo rats respectively that belong to the tribe Otomyini and are both endemic to Africa. They are diurnal, exclusively herbivorous and occupy arid and semi-arid habitats for P. brantsii and semi-arid Karoo habitats in the case of O. unisculcatus. Parotomys brantsii is considered solitary; its reproduction is opportunistic, and dependent on rainfall, a gestation length of 38 days, a life span of 1-2 years anda mean litter size of 3.4. Otomys unisulcatus is considered social with an opportunistic reproduction that is dependent on rainfall. Its gestation length is 37 days with a mean litter size of 2.09 and a life span of 1-2 years. Natal multimammate mouse Mastomys natalensis is endemic to Africa and occurs widely across Sub-Saharan Africa where it is typically associated with agricultural fields and houses but also natural savannas and grasslands. It is one of the commonest species in savanna habitats. It is nocturnal and an opportunistic omnivore. It is particularly commensal in some parts of West and Central Africa and has been closely associated as a reservoir with the spread of Lassa fever in these areas. Reproduction is very seasonal, with a mean litter-size of 6.5, a gestation that lasts 21-22 days, a life span of less than 12 months and it is considered social. Micaelamys namaquensis is also endemic to Africa, nocturnal, semi-arboreal and an opportunistic omnivore. This species is widely distributed in Southern Africa occurring in a variety of habitats, but usually associated with rocky outcrops in savanna and semi-arid areas. It is reported as nesting singly, in pairs and small groups with a gestation length that is greater than 22 days, a life span of 12 months, mean litter-size of 3.3, and a seasonal reproduction that is confined to wet season (Denys et al., 2017; Happold, 2013; Monadjem et al., 2015).
